# Unexpected Stroke Two Years After Atrial Septal Defect Closure: A Case of Late Device Embolization

**DOI:** 10.1002/ccr3.70718

**Published:** 2025-08-14

**Authors:** Nuria Pueyo‐Balsells, Ignacio Barriuso, Marta Zielonka, Carlos Izurieta, Kristian Rivera

**Affiliations:** ^1^ Department of Cardiology Arnau de Vilanova University Hospital Lleida Spain; ^2^ University Hospital San Agustin Avilés Spain; ^3^ Grup de Fisiologia i Patologia Cardíaca, Institut de Recerca Biomèdica de Lleida Fundació Dr. Pifarré IRBLleida Lleida Spain

**Keywords:** atrial septal defect, device embolization, ischemic stroke, multimodality imaging, thrombophilia, transcatheter closure

## Abstract

A 42‐year‐old woman with a C677T mutation in the methylenetetrahydrofolate reductase gene and a history of transient ischemic attack underwent transcatheter closure of an ostium secundum atrial septal defect (ASD‐OS) using a Figulla Flex II device. Two years later, she presented with an ischemic stroke. Imaging revealed late embolization of the occluder device to the aorto‐iliac bifurcation. As she remained asymptomatic, a conservative approach was adopted. A second transcatheter ASD‐OS closure was successfully performed. This case underscores the potential contribution of inherited thrombophilia to recurrent embolic events, the importance of long‐term surveillance for device complications, and the value of multimodal imaging in guiding clinical decision making.

**Summary**

- Late embolization of atrial septal occluder devices can present as stroke years after implantation, particularly in patients with underlying thrombophilia.
- Multimodal imaging is essential for diagnosis.
- In asymptomatic cases, conservative management may be appropriate, highlighting the importance of personalized care and long‐term follow‐up in structural heart disease.

## 1 Case Presentation

**FIGURE 1:**
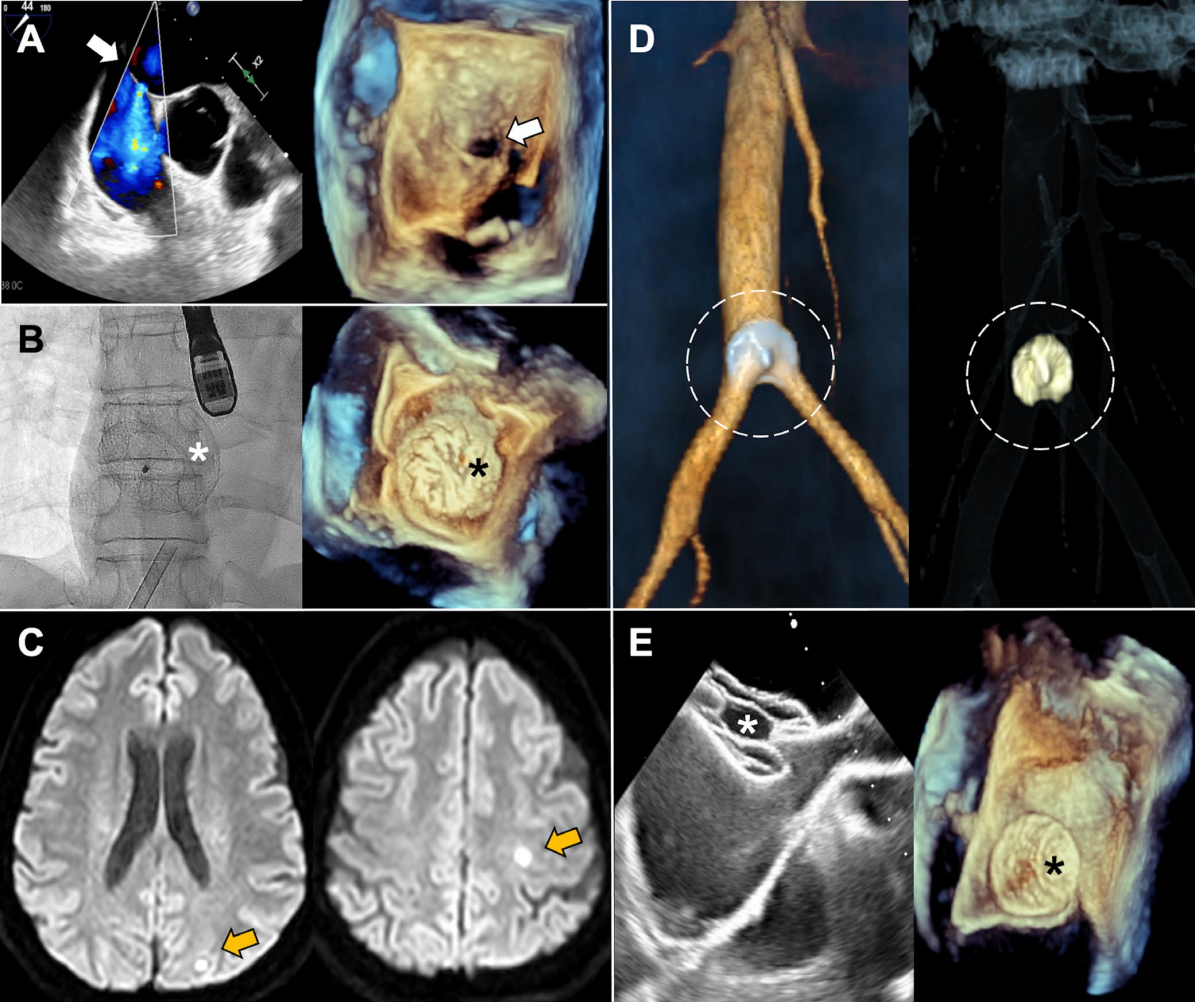
(A) Left‐to‐right shunt across an ASD‐OS seen on the TEE color Doppler (white arrow, left) and 3D‐TEE (white arrow, right). (B) First device implantation: 17 mm Figulla Flex II UNI seen by fluoroscopy (white asterisk) and 3D‐TEE (black asterisk). (C) Brain MRI showing two acute lacunar infarcts with restricted diffusion (orange arrows) in the left occipital lobe and the precentral gyrus. (D) 3D‐CT reconstruction showing the embolized occluder at the aorto‐iliac bifurcation (dashed circle). (E) Second device implantation: 16 mm Occlutech 29ASD visualized by TEE (white and black asterisks). 3D‐CT, three‐dimensional computed tomography; ASD‐OS, ostium secundum atrial septal defect; MRI, magnetic resonance imaging; TEE, transesophageal echocardiography.

A 42‐year‐old woman with a known C677T mutation in the methylenetetrahydrofolate reductase (MTHFR) gene presented with a transient ischemic attack (TIA) 2 years earlier. Transesophageal echocardiography (TEE) revealed an ostium secundum atrial septal defect (ASD‐OS) with a 4 mm left‐to‐right shunt (Figure 1A). Given the suspected paradoxical embolism in the context of thrombophilia, transcatheter closure was performed using a 17 mm Figulla Flex II UNI device (Occlutech, Sweden) (Figure 1B). Two years later, she presented with an ischemic stroke. Diffusion‐weighted magnetic resonance imaging (MRI) demonstrated two acute subcentimeter lacunar infarcts: one cortico‐subcortical in the left precentral gyrus and another subcortical in the postero‐superior occipital pole (Figure 1C). Repeat TEE revealed a recurrent left‐to‐right shunt and absence of the occluder in the atrial septum, with no evidence of thrombus or vegetation. Thoracoabdominal computed tomography angiography (CTA) identified the embolized device lodged at the aorto‐iliac bifurcation (Figure 1D). Nevertheless, the patient remained asymptomatic, with no signs of lower limb ischemia or decreased pulses. Following multidisciplinary discussion, a conservative approach to the embolized device was adopted. A second ASD‐OS closure was performed without complications, using a 16 mm Occlutech 29ASD device (Occlutech, Sweden) (Figure 1E). At one‐year follow‐up, the patient continued to be asymptomatic with no recurrent neurological events or residual shunt.

## 2 Discussion

This case highlights several key insights. First, inherited thrombophilia, such as the MTHFR C677T mutation, should be considered in the etiological evaluation of cryptogenic stroke in younger patients. Thrombophilia screening reveals abnormalities in over 20% of patients with TIA or ischemic stroke [1]. Although the clinical impact of isolated variants of MTHFR remains debated, they may act synergistically with structural heart defects such as ASD or patent foramen ovale to increase embolic risk [1, 2]. In such cases, percutaneous closure can be justified, even when the annual recurrence risk of stroke from ASD is relatively low (~0.6%) [2]. Second, device embolization is a rare but serious complication, with an estimated incidence of 0%–3.9% per 100 person‐years, particularly in cases involving large or multiple devices [3]. Although Figulla Flex II has demonstrated a low embolization rate and is preferred in challenging anatomies, late embolization, even years after implantation, should be considered in patients with recurrent embolic events. Third, this case highlights the value of multimodal imaging, not only for diagnosis but also to guide treatment decisions. Finally, in asymptomatic patients with embolized devices and no signs of organ ischemia, conservative management may be appropriate. An individualized risk–benefit assessment is essential, balancing the risks of surgical retrieval or intervention against the potential for stable long‐term outcomes. Continued follow‐up is crucial to ensure safety over time.

## Author Contributions

**Nuria Pueyo‐Balsells:** conceptualization, data curation, formal analysis, investigation, methodology, project administration, resources, software, supervision, validation, visualization, writing – original draft, writing – review and editing. **Ignacio Barriuso:** data curation, formal analysis, investigation, methodology, resources, software, supervision, validation, visualization, writing – original draft, writing – review and editing. **Marta Zielonka:** data curation, formal analysis, investigation, methodology, resources, software, supervision, validation, visualization, writing – original draft, writing – review and editing. **Carlos Izurieta:** data curation, formal analysis, investigation, methodology, resources, software, supervision, validation, visualization, writing – original draft, writing – review and editing. **Kristian Rivera:** data curation, formal analysis, investigation, methodology, resources, software, supervision, validation, visualization, writing – original draft, writing – review and editing.

## Ethics Statement

This article was conducted according to the principles of the Declaration of Helsinki.

## Consent

Written informed consent was obtained from the patient for the publication of this article in accordance with the patient consent policy of the journal.

## Conflicts of Interest

The authors declare no conflicts of interest.

## Data Availability

All generated data are included in this article.

## References

[1] B. Sánchez‐Marín and J. M. Grasa , “Methylenetetrahydrofolate Reductase (MTHFR) C677T Polymorphism in Ischemic Vascular Disease,” Revista de Neurologia 43 (2006): 630–636. 17099857

[2] S. Eppinger , K. Piayda , R. Galea , et al., “Embolization of Percutaneous Left Atrial Appendage Closure Devices: Timing, Management and Clinical Outcomes,” Cardiovascular Revascularization Medicine 64 (2024): 7–14. 38448258 10.1016/j.carrev.2024.02.014

[3] S. T. Lim , S. J. X. Murphy , D. R. Smith , et al., “Clinical Outcomes and a High Prevalence of Abnormalities on Comprehensive Arterial and Venous Thrombophilia Screening in TIA or Ischaemic Stroke Patients With a Patent Foramen Ovale, an Inter‐Atrial Septal Aneurysm or Both,” Journal of the Neurological Sciences 15 (2017): 227–233. 10.1016/j.jns.2017.04.014 28477701

